# Surgical-orthodontic treatment for class II asymmetry: outcome and influencing factors

**DOI:** 10.1038/s41598-019-54317-5

**Published:** 2019-11-29

**Authors:** Yun-Fang Chen, Yu-Fang Liao, Ying-An Chen, Yu-Ray Chen

**Affiliations:** 1 Department of Craniofacial Orthodontics, Chang Gung Memorial Hospital, Taipei, Taiwan; 2 Craniofacial Center, Chang Gung Memorial Hospital, Taoyuan, Taiwan; 3 Craniofacial Research Center, Chang Gung Memorial Hospital, Linkou, Taiwan; 4 Graduate Institute of Dental and Craniofacial Science, Chang Gung University, Taoyuan, Taiwan; 5 Department of Craniofacial Orthodontics, Chang Gung Memorial Hospital, Taoyuan, Taiwan; 6 Department of Plastic and Reconstructive Surgery, Chang Gung Memorial Hospital, Linkou, Taiwan

**Keywords:** Risk factors, Reconstruction

## Abstract

The study aimed to evaluate the treatment outcome of bimaxillary surgery for class II asymmetry and find the influencing factors for residual asymmetry. Cone-beam computed tomographic images of 30 adults who had bimaxillary surgery were acquired, and midline and contour landmarks of soft tissue and teeth were identified to assess treatment changes and outcome of facial asymmetry. The postoperative positional asymmetry of each osteotomy segment was also measured. After surgery, the facial midline asymmetry of the mandible, chin, and lower incisors improved significantly (all p < 0.01). However, the residual chin deviation remained as high as 2.64 ± 1.80 mm, and the influencing factors were residual shift asymmetry of the mandible (p < 0.001), chin (p < 0.001), and ramus (p = 0.001). The facial contour asymmetry was not significantly improved after surgery, and the influencing factors were the initial contour asymmetry (p < 0.001), and the residual ramus roll (p < 0.001) or yaw (p < 0.01) asymmetry. The results showed that bimaxillary surgery significantly improved midline but not contour symmetry. The postoperative midline and contour asymmetry was mainly affected by the residual shift and rotational jaw asymmetry respectively.

## Introduction

Facial asymmetry is commonly seen in adults. The causes of facial asymmetry include skeletal asymmetry, soft tissue asymmetry, functional asymmetry or a combination^1^. Of these, skeletal asymmetry involving deviations in the maxillofacial region is predominant. In instances of jaw asymmetry, distinctions in type of asymmetry can be further divided into positional, size, and shape asymmetry^2,3^. Positional asymmetry of the jaw can be further described into shift (translation), roll or yaw (rotation) asymmetry^4^.

Positional asymmetry of the jaw must be addressed before size and shape asymmetry can be efficiently diagnosed and corrected and the only procedure for centering jaws in adult patients is orthognathic surgery (OGS). Many studies on correcting facial asymmetry with OGS have been conducted, however most corrections were on patients with class III deformity^5–14^ due to the lower incidence and extent of facial asymmetry in class II deformity^15–17^. Furthermore, the focus of most of these studies was on improvement of midline asymmetry. Symmetry of gonial width improved after OGS in a study by Ko *et al*. and frontal ramal symmetry improved for many patients in a study by Chen *et al*.; however, their investigation of the change of contour asymmetry was limited to skeletal structures^8,14^. Despite Blockhaus *et al*. and Hajeer *et al*. studying outcomes of OGS on patients with class II asymmetry, there was a lack of information regarding contour asymmetry^16,18^. Importantly, none of the above studies analyzed the impact of positional jaw asymmetry on residual asymmetry with regards to soft tissue and dental aspects.

The introduction and development of cone-beam computed tomography (CBCT) makes the accurate evaluation of the 3-dimensional (3D) complexity of facial asymmetry possible. Therefore, this study aimed to evaluate the asymmetry outcome (midline and contour symmetry) of bimaxillary OGS for class II asymmetry and find the influencing factors for residual asymmetry.

## Results

Thirty patients with class II asymmetry (9 men and 21 women; mean age, 29.3 ± 5.6 years; range, 19.0 to 47.0 years) fulfilled the inclusion criteria and were analyzed. The postoperative CBCT was taken 20.6 ± 7.2 months after surgery, on average (range, 12.0 to 34.6 months). The bimaxillary surgery was highly effective for class II deformity with significant improvement in the ANB angle (from 7.2 ± 2.0 degrees to 3.7 ± 1.5 degrees, p < 0.001) and overjet (from 4.8 ± 3.0 mm to 2.7 ± 0.6 mm, p < 0.01).

### Asymmetry outcome

The bimaxillary surgery significantly improved the midline deviation of the mandible (p < 0.01), chin (p < 0.001), and lower incisors (p < 0.01). Nevertheless, the midline deviation of the nose deteriorated after surgery (p < 0.001). There was no significant improvement in the lip cant, upper, middle, or lower contour asymmetry (p > 0.01) (Table 1).

**Table 1:** Facial asymmetry^a^ before and after bimaxillary surgery.

Facial asymmetry	Before		After		p
	Mean	SD	Mean	SD	
Soft tissue asymmetry					
Nasal midline deviation (mm)	0.34	0.79	0.81	0.94	<0.001
Upper lip midline deviation (mm)	0.79	0.68	1.02	0.85	0.071
Lower lip midline deviation (mm)	1.73	1.29	1.31	1.14	0.164
Mandibular midline deviation (mm)	3.13	2.13	1.88	1.26	0.002
Chin midline deviation (mm)	5.61	3.27	2.64	1.80	<0.001
Lip cant (mm)	1.73	1.19	1.09	0.87	0.013
Upper contour asymmetry (mm)	4.64	4.07	4.17	3.26	0.543
Middle contour asymmetry (mm)	5.99	4.85	4.90	3.62	0.270
Lower contour asymmetry (mm)	6.86	5.24	5.15	3.67	0.139
Dental asymmetry					
Upper dental midline deviation (mm)	1.12	0.82	1.71	1.54	0.054
Lower dental midline deviation (mm)	2.66	1.70	1.75	1.51	0.008

^a^Absolute values were used to present the extent of facial asymmetry.

### Influencing factors for residual asymmetry

Stepwise multiple linear regression analysis demonstrated the residual midline deviation of the nose and upper lip was associated with the initial deviation and the residual maxillary shift asymmetry (all p ≤ 0.001). The residual midline deviation of the lower lip was associated with the residual mandibular shift asymmetry (p = 0.001). The residual midline deviation of the mandible was affected by the residual shift and yaw asymmetry of the mandible (both p < 0.001). The residual midline deviation of the chin was affected by the residual shift asymmetry of the mandible (p < 0.001), chin (p < 0.001), and ramus (p = 0.001). The residual lip cant was affected by the residual mandibular roll asymmetry (p < 0.001) (Table 2).

**Table 2:** Multiple linear regression analysis: influencing factors for postoperative facial asymmetry.

Dependent variable	Model	B	SE	p	R^2^
Nasal midline deviation	(Constant)	0.064	0.139	0.649	0.656
	Initial nasal midline deviation	0.941	0.164	<0.001	
	Maxillary shift asymmetry	0.456	0.154	<0.001	
Upper lip midline deviation	(Constant)	0.112	0.169	0.515	0.560
	Maxillary shift asymmetry	0.672	0.129	<0.001	
	Initial upper lip midline deviation	0.611	0.166	0.001	
Lower lip midline deviation	(Constant)	0.416	0.297	0.172	0.327
	Mandibular shift asymmetry	0.426	0.116	0.001	
Mandibular midline deviation	(Constant)	0.172	0.149	0.258	0.900
	Mandibular shift asymmetry	0.769	0.060	<0.001	
	Mandibular yaw asymmetry	0.220	0.051	<0.001	
Chin midline deviation	(Constant)	0.342	0.206	0.109	0.911
	Mandibular shift asymmetry	0.760	0.111	<0.001	
	Chin shift asymmetry	0.482	0.087	<0.001	
	Ramus shift asymmetry	−0.242	0.066	0.001	
Lip cant	(Constant)	0.570	0.199	0.008	0.564
	Mandibular roll asymmetry	0.444	0.074	<0.001	
Upper contour asymmetry	(Constant)	0.710	0.560	0.216	0.686
	Initial upper contour asymmetry	0.500	0.094	<0.001	
	Ramus roll asymmetry	0.431	0.431	<0.001	
Middle contour asymmetry	(Constant)	1.164	0.612	0.068	0.734
	Initial middle contour asymmetry	0.465	0.084	<0.001	
	Ramus roll asymmetry	0.633	0.119	<0.001	
	Ramus yaw asymmetry	0.433	0.150	0.008	
Lower contour asymmetry	(Constant)	1.646	0.661	0.019	0.704
	Ramus roll asymmetry	0.690	0.127	<0.001	
	Ramus yaw asymmetry	0.473	0.162	0.007	
	Initial lower contour asymmetry	0.396	0.080	<0.001	
Upper dental midline deviation	(Constant)	−0.928	0.263	0.002	0.592
	Maxillary shift asymmetry	0.655	0.225	0.007	
	Mandibular yaw asymmetry	0.441	0.104	<0.001	
Lower dental midline deviation	(Constant)	−0.426	0.317	0.191	0.556
	Mandibular shift asymmetry	0.421	0.128	0.003	
	Mandibular yaw asymmetry	0.387	0.105	0.001	

B, unstandardized regression coefficient; SE, standard error.

The residual upper contour asymmetry was associated with its initial asymmetry and the residual ramus roll asymmetry (both p < 0.001). The residual middle and lower contour asymmetry was associated with their initial asymmetry (both p < 0.001) and the residual ramus roll (both p < 0.001) and yaw asymmetry (both p < 0.01) (Table 2).

The residual upper dental midline deviation was affected by the residual mandibular yaw asymmetry (p < 0.001) and maxillary shift asymmetry (p < 0.01). The residual lower dental midline deviation was affected by the residual yaw (p = 0.001) and shift (p < 0.01) asymmetry of the mandible (Table 2).

## Discussion

Patient satisfaction for correction of sagittal deformity and malocclusion via OGS requires improvement of facial asymmetry. Improvement of midline asymmetry of soft tissue and incisors shown in the frontal view, including midline deviation and lip cant, are of primary importance for patients to assess an asymmetry outcome positively^6,7,19^. Nevertheless, the frontal contour asymmetry, which is indeed altered by OGS and is of great clinical relevance^20^, has long been overlooked. Thus, the facial landmarks chosen in the present study covered midline and contour regions, intending to provide a measuring technique which is easily applicable and practical for clinicians to assess facial symmetry during and after operation.

This study also evaluated the underlying jaw characteristics contributing to the residual asymmetry after surgery. After bimaxillary surgery, the extent of residual facial asymmetry was highest in the lower contour region, and decreased in the order of middle and upper contour, chin, mandible, lower and upper incisors, lower lip, lip commissures, upper lip, and nose. The trend was almost the same as preoperative asymmetry (Table 1). For the contour region, the asymmetry was improved via surgery but not well enough to reach significance, which was correlated with the initial asymmetry and the roll or yaw asymmetry of the ramus (Table 2).

Although the mandibular body is the underlying skeletal support corresponding to the soft tissue envelope of the lower contour region, there was no significant correlation in between. Residual lower contour asymmetry was directly affected by the ramus, rather than the underlying mandibular body. One possible explanation is the modified Hunsuck technique, which extends the anterior cut of the osteotomy to the first molar. Another explanation is the limitation of the roll rotation of the proximal segment during surgery^14^. This speculation is supported by the significant correlation between the postoperative ramus roll asymmetry and the postoperative mandibular yaw asymmetry (r = −0.61, p < 0.001).

The threshold of clinical acceptance for midline asymmetry has been reported to be approximately 2 mm, including the upper dental midline^21,22^, lower dental midline^21^, and chin midline^2,21,23^. Thus, the results in Table 1 showing the treatment outcome of soft tissue midline asymmetry, with the exception of the chin, was favorable when the mean value was ≤2 mm. Although the soft tissue chin midline deviation in skeletal class II showed the greatest improvement (2.97 mm, 52.9%, p < 0.001), noticeable asymmetry (2.64 ± 1.80 mm) was still observed after surgery. This finding of residual chin deviation is consistent with previous OGS studies on different types of malocclusion^7–9,12,16^, suggesting the difficulty in the recognition of facial midline intra-operatively or relapse post-operatively. The present study provided further evidence that the residual chin asymmetry is correlated with the residual shift asymmetry of the mandible, chin, and ramus (Table 2).

The lip cant was insignificantly improved after bimaxillary OGS (0.64 mm, 37.0%, p = 0.013). We found that the mandibular roll asymmetry, rather than the maxillary roll asymmetry, affected the lip cant postoperatively (Table 2). In addition, postoperative mandibular roll asymmetry was not necessarily correlated with postoperative maxillary roll asymmetry (r = 0.35, p = 0.055). In the study by Suzuki-Okumara *et al*., the preoperative measurements showed the same correlation, in which the preoperative lip cant was correlated with the preoperative mandibular roll asymmetry rather than the maxillary roll asymmetry^12^. Interestingly, Suzuki-Okumara *et al*., also reported that the change of lip cant was correlated with the change of the maxillary roll asymmetry, rather than the change of the mandibular roll asymmetry^12^. Although the present study did not measure the change in roll asymmetry of the maxilla or mandible, many of the OGS studies on lip cant consistently found a significant correlation between the change of lip cant and the change of the maxillary occlusal cant^6,10,19^. The study by Kim *et al*. demonstrated the average amount of lip cant correction was approximately 50% of the maxillary occlusal cant correction^19^. Therefore, in addition to correction of maxillary occlusal cant, correction of mandibular roll asymmetry might also play a role in further restoring lip symmetry.

Residual maxillary and mandibular shift asymmetry were found to be the most important factors influencing the postoperative upper and lower dental midline deviation, respectively. Residual mandibular yaw asymmetry also affected the upper and lower dental midline deviation postoperatively. Residual maxillary yaw asymmetry was found to have no significant influence on residual upper dental midline asymmetry, although mandibular yaw asymmetry was significantly correlated with maxillary yaw asymmetry (r = 0.63, p < 0.001). Song *et al*. analyzed pre-treatment variables and found that maxillary yaw asymmetry was the primary contributing factor for upper dental midline deviation in patients with upper dental midline deviation greater than 2 mm; however, analysis of mandibular variables was not shown^22^. Ryu *et al*. also analyzed pre-treatment variables and found that upper dental midline deviation was not significantly correlated with mandibular roll or yaw asymmetry, and lower dental midline deviation was significantly correlated with mandibular yaw asymmetry^24^. The possible reasons for the discordant findings about influencing factors for upper dental midline deviation include heterogeneity of samples (class III asymmetry vs. class II asymmetry), different variables for positional jaw asymmetry (line vs. plane), and the intervention of surgical-orthodontic treatment (no vs. yes).

The midline deviation of the nose became more severe after surgery (Table 1). This could be explained by the shift asymmetry of the maxilla after surgery (Table 2). Sacrifice of the nasal and maxillary symmetry has also been shown to achieve favorable mandibular and overall facial symmetry^25^. Despite the deterioration, the mean residual midline deviation of the nose was less than 1 mm, which is usually clinically acceptable^2,21,22^.

This study has some limitations. First, the degree of facial asymmetry in patients with class II deformities is usually modestly remarkable and consequently the sample size of this study was small. Studies with larger sample size are needed to draw more robust conclusions. Second, the occlusal plane cant, which might play an important role on mandibular shift and chin deviation, was not measured. Finally, no size influence of soft tissue or jaws was analyzed. Future studies are needed to explore the impact of occlusal cant, soft and hard tissue volume on the treatment outcome of facial asymmetry.

## Conclusions

The findings of the present study showed bimaxillary OGS for patients with class II asymmetry significantly improved the midline asymmetry of the chin, mandible, and lower incisors. However, noticeable chin deviation was still observed after surgery, which was affected by the residual shift asymmetry of the mandible, chin, and ramus. The contour asymmetry was not significantly improved after surgery, which was affected by the initial severity of contour asymmetry, and the residual roll or yaw asymmetry of the ramus. Maxillary shift asymmetry was the primary factor influencing the postoperative midline deviation of the nose, upper lip, and upper incisors. Mandibular shift asymmetry was the primary factor influencing the postoperative midline deviation of the lower lip, mandible, chin, and lower incisors.

## Material and Methods

### Patients

The retrospective study was conducted in accordance with the World Medical Association Declaration of Helsinki on medical research ethics. The approval of the study was granted by the Ethics Committee for Human Research at the Chang Gung Memorial Hospital in Taoyuan, Taiwan. The need for informed consent was waived by the Ethics Committee that approved the study due to the retrospective design of the study. Thirty Taiwanese adults (age ≥18 years) with class II deformity (A point–nasion–B point angle >4 degrees) and significant facial asymmetry (skeletal menton deviation >2 mm or lip cant >2 mm or significant contour asymmetry) were selected based on the following criteria: (1) consecutive Le Fort I osteotomy and bilateral sagittal split osteotomy (BSSO) advancement surgery by the attending surgeons supervised by one senior surgeon with more than 40 years of experience at the Chang Gung Craniofacial Center during a 3-year period, (2) completion of postsurgical orthodontic treatment, (3) no progressive or chronic temporomandibular joint disorder, (4) no other craniofacial deformities or genetic syndromes, (5) no history of craniofacial surgery or trauma, and (6) available CBCT taken at two time points, before surgery and at least 12 months after surgery, on the day of orthodontic debonding. The informed consent for publication of identifying information/images in an online open-access publication was obtained from the patient whose images were displayed.

### Surgical technique

The BSSO was modified from Hunsuck^26^ by extending the anterior cut of the osteotomy to the first molar^27,28^. The Le Fort I osteotomy was performed with a technique similar to that popularized by Bell^29^. No additional surgical intervention other than genioplasty was performed. Rigid fixation was performed with bone plates or screws. On average, the anterior maxilla (incisive foramen) moved backward (1.16 mm), upward (2.36 mm), and toward the opposite side (opposite to menton-deviated side, 0.23 mm). The posterior maxilla (greater palatine foramen) moved forward (0.49 mm and 0.61 mm, respectively for the deviated and opposite sides), upward (0.74 mm and 1.93 mm, respectively for the deviated and opposite sides), and toward the opposite side (0.11 mm and 0.25 mm, respectively for the deviated and opposite sides). The anterior mandible (genial tubercle) moved forward (3.87 mm) and toward the opposite side (2.13 mm). The posterior mandible (mental foramen) moved forward (2.86 mm and 3.04 mm, respectively for the deviated and opposite sides), downward (2.31 mm and 0.95 mm, respectively for the deviated and opposite sides), and toward the opposite side (2.23 mm and 1.28 mm, respectively for the deviated and opposite sides).

### CBCT

CBCT of the head and neck was performed using an i-CAT 3D Dental Imaging System (Imaging Sciences International, Hatfield, PA, USA) with the following parameters: 120 kVp, 0.4 mm × 0.4 mm × 0.4 mm voxel size, 40 second scan time, and 16 cm × 16 cm field of view. The patient’s head was positioned with the Frankfort horizontal plane parallel to the ground. Throughout the scan, patients were asked not to swallow.

**Figure 1:**
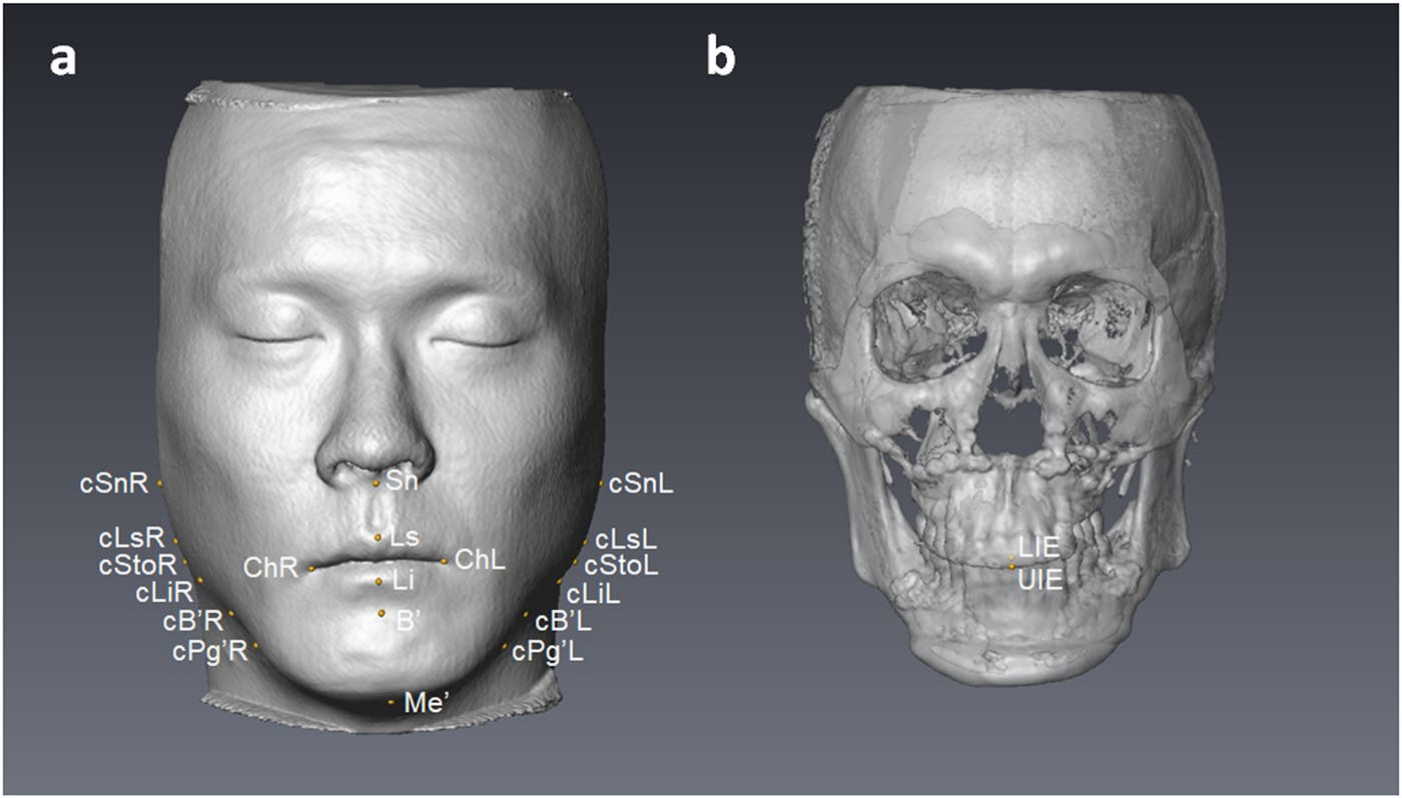
Landmarks used for asymmetry outcome analysis. (**a**) Soft tissue landmarks: Sn, subnasale; Ls, labiale superius; Li, labiale inferius; B’, soft tissue B point; Me’, soft tissue menton; ChR, cheilion right; ChL, cheilion left; cSnR, contour Sn point right; cSnL, contour Sn point left; cLsR, contour Ls point right; cLsL, contour Ls point left; cStoR, contour Sto point right; cStoL, contour Sto point left; cLiR, contour Li point right; cLiL, contour Li point left; cB’R, contour B’ point right; cB’L, contour B’ point left; cPg’R, contour Pg’ point right; cPg’L, contour Pg’ point left. (**b**) Dental landmarks: UIE, upper incisal embrasure; LIE, lower incisal embrasure.

**Figure 2:**
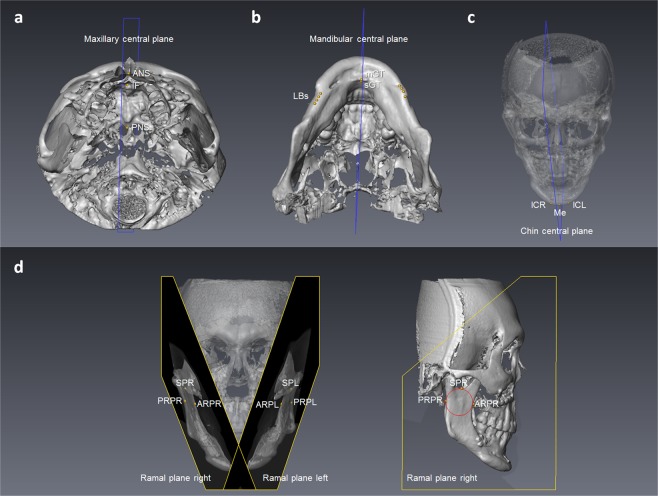
Landmarks and planes used for positional jaw asymmetry analysis. (**a**) Maxillary central plane: ANS, anterior nasal spine; IF, incisive foramen; PNS, posterior nasal spine. (**b**) Mandibular central plane: mGT, genial tubercle midpoint; sGT, genial tubercle superius; LBs, lower border points. (**c**) Chin central plane: Me, menton; lCR, lateral chin point right; lCL, lateral chin point left. (**d**) Ramal plane right: SPR, sigmoid point right; ARPR, anterior ramal point right; PRPR, posterior ramal point right. Ramal plane left: SPL, sigmoid point left; ARPL, anterior ramal point left; PRPL, posterior ramal point left. The inscribed circle (red) within the ramus was used to define the tangents of SPR, ARPR and PRPR.

Images were stored in the Digital Imaging and Communications in Medicine (DICOM) format and then transferred to a workstation (Avizo v7.0.0 software, FEI, Mérignac, France) where they were rendered into volumetric images, segmented and analyzed by one single investigator (CYF) blinded to the patients’ treatment histories. Before analysis, six skeletal landmarks were selected for registration of the 3D images in a 3D coordinate system (x, y, z) given in millimeters with nasion as the zero point: nasion, bilateral porion, bilateral orbitale, and basion. The horizontal reference plane was parallel with the FH plane (the best-fit plane passing through bilateral porion and orbitale) and passing through nasion. The midsagittal plane was perpendicular to the horizontal reference plane and passing through nasion and basion. The coronal reference plane was perpendicular to the horizontal and midsagittal reference planes and passing through nasion. A positive value indicates the left, posterior and superior side of the face. After registration of the 3D images, landmarks^30–32^ and planes used for measurement (Tables 3 and 4, and Figs. 1 and 2) were located on the 3D surface models by the same investigator. Multiplanar reconstruction views were also used to identify the landmarks when necessary.

**Table 3:** Landmarks used for analysis of treatment outcome.

Landmarks	Symbol	Definition
Soft tissue		
Midline		
Subnasale	Sn	The midpoint on the nasolabial soft tissue contour between the columella crest and the upper lip
Labiale superius	Ls	The midpoint of the vermilion line of the upper lip
Labiale inferius	Li	The midpoint of the vermilion line of the lower lip
Soft tissue B point	B’	The most posterior midpoint on the labiomental soft tissue contour that defines border between the lower lip and the chin
Soft tissue menton	Me’	The most inferior midpoint on the soft tissue contour of the chin
Cheilion	Ch	The point located at each labial commissure
Contour		
Contour Sn point	cSn	The intersection point formed by the most lateral point on the face and the line parallel to the horizontal reference plane passing through Sn
Contour Ls point	cLs	The intersection point formed by the most lateral point on the face and the line parallel to the horizontal reference plane passing through Ls
Contour Sto point	cSto	The intersection point formed by the most lateral point on the face and the line parallel to the horizontal reference plane passing through Sto^a^
Contour Li point	cLi	The intersection point formed by the most lateral point on the face and the line parallel to the horizontal reference plane passing through Li
Contour B’ point	cB’	The intersection point formed by the most lateral point on the face and the line parallel to the horizontal reference plane passing through B’
Contour Pg’ point	cPg’	The intersection point formed by the most lateral point on the face and the line parallel to the horizontal reference plane passing through Pg’^b^
Teeth		
Midline		
Upper incisal embrasure	UIE	The incisal embrasure between the upper central incisors
Lower incisal embrasure	LIE	The incisal embrasure between the lower central incisors

^a^Stomion, the midpoint of the horizontal labial fissure.

^b^Soft tissue pogonion, the most anterior midpoint of the chin.

**Table 4:** Landmarks and planes used for analysis of positional jaw asymmetry.

Landmarks	Symbol	Definition
Maxilla		
Anterior nasal spine	ANS	The most anterior midpoint of the anterior nasal spine of the palatine bone
Posterior nasal spine	PNS	The most posterior midpoint of the posterior nasal spine of the palatine bone
Incisive foramen	IF	The most posterior midpoint of the incisive foramen
Mandible (mandibular body)		
Genial tubercle superius	sGT	The most superior midpoint of all genial tubercles
Genial tubercle midpoint	mGT	The center point of all genial tubercles
Lower border points	LBs	The most inferior points of the lower border of the mandibular body on a series of four coronal planes of 3 mm interval from the distal end of genioplasty^a^
Chin		
Menton	Me	The most inferior midpoint of the chin on the outline of the mandibular symphysis
Lateral chin point	lC	The most inferior and lateral point on each angle of the chin
Ramus		
Sigmoid point	SP	The tangent point between the sigmoid notch and the inscribed circle within the ramus
Anterior ramal point	ARP	The tangent point between the anterior border of the ramus and the inscribed circle within the ramus
Posterior ramal point	PRP	The tangent point between the posterior border of the ramus and the inscribed circle within the ramus
Planes		
Maxillary central plane	MxCP	A plane passing through ANS, PNS, and IF
Mandibular central plane	MdCP	The best fit plane of sGT and the line bisecting the angle formed by the two least squares lines of bilateral LBs, passing through mGT
Chin central plane	ChinCP	A plane perpendicular to the line connecting bilateral lC, and passing through Me
Ramal plane	RP	A plane passing through SP, ARP, and PRP

^a^For the two OGS patients without genioplasty, the designation of LBs were started from the sagittal level of the mental foramen.

### Asymmetry outcome

To demonstrate the extent of asymmetry before and after surgery, absolute values of the following measurements for soft tissue and dental asymmetry were used. Real values were used for regression analysis.

### Soft tissue asymmetry: midline and contour asymmetry

1. Midline asymmetry:
  - Midline deviation: the transverse distances between midline landmarks of Sn, Ls, Li, B’, Me’ and the midsagittal plane (MSP) were measured as the midline deviation of the nose, upper lip, lower lip, mandible, and chin, respectively (Table 3).
  - Lip cant: the vertical distance between bilateral landmarks of Ch was measured as the lip cant.
2. Contour asymmetry: the discrepancy in transverse distances of bilateral contour landmarks at the same level from the MSP
  - Upper contour asymmetry: the summation of contour asymmetry at Sn level and Ls level.
  - Middle contour asymmetry: the summation of contour asymmetry at Sto level and Li level.
  - Lower contour asymmetry: the summation of contour asymmetry at B’ level and Pg’ level.

### Dental asymmetry: midline asymmetry

1. Midline asymmetry:
  - Midline deviation: the transverse distances between the midline landmarks of UIE and LIE and the MSP were measured as the midline deviation of the upper incisors and lower incisors (Table 3).

### Influencing factors

**Figure 3:**
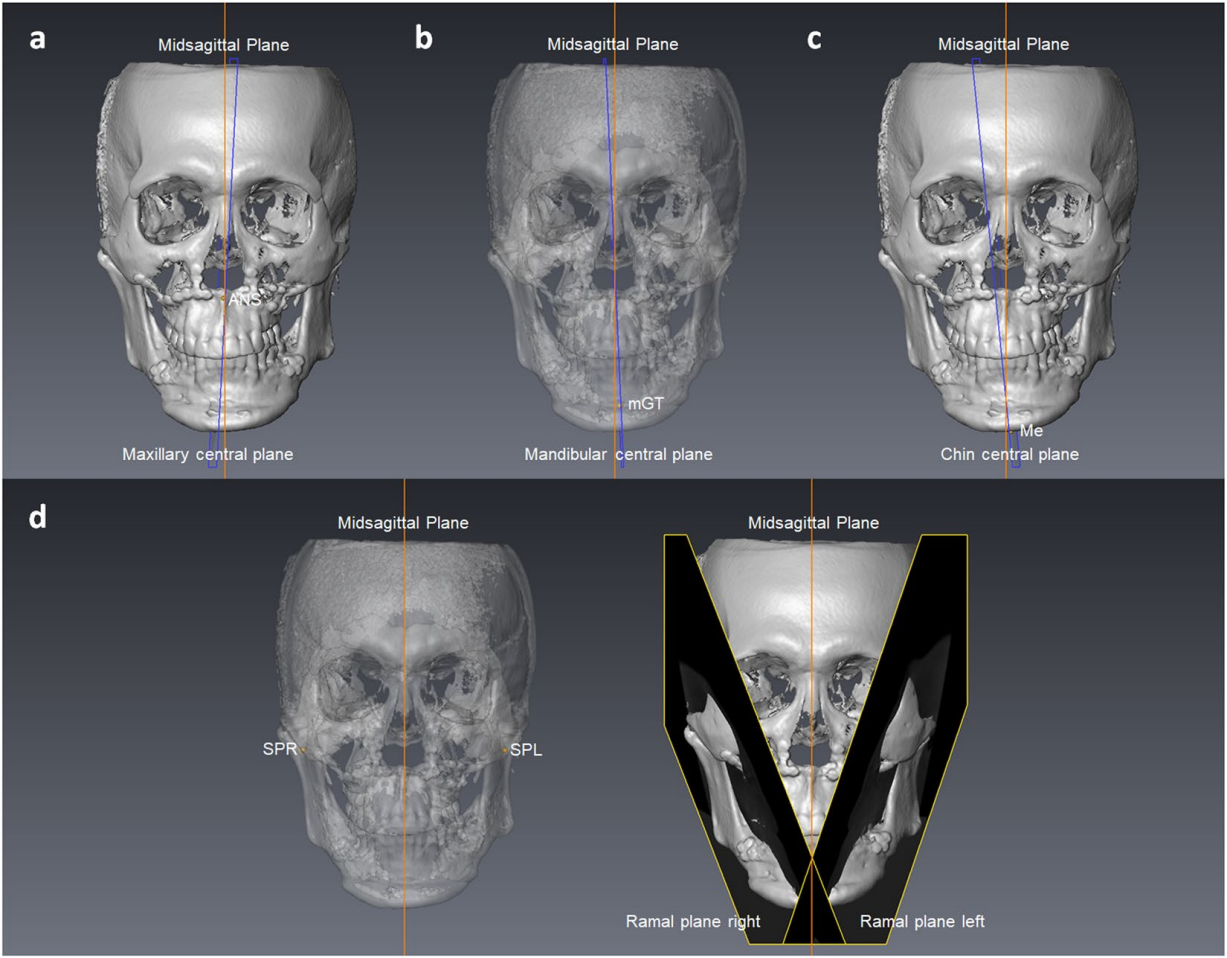
Example of a patient showing positional jaw asymmetry after bimaxillary surgery. (**a**) Maxilla: ANS, anterior nasal spine (**b**) Mandible: mGT, genial tubercle midpoint. (**c**) Chin: Me, menton. (**d**) Ramus: SPR, sigmoid point right; SPL, sigmoid point left.

The possible influencing factors for residual asymmetry included preoperative asymmetry and postoperative positional jaw asymmetry in terms of shift, roll or yaw asymmetry. To evaluate the positional jaw asymmetry, we first defined five planes (maxillary central plane, mandibular central plane, chin central plane, and bilateral ramal planes) for each osteotomy segment (Table 4). Then, the discrepancy of the five planes from the reference planes was calculated to quantify the shift, roll or yaw asymmetry of the maxilla, mandible, chin and ramus (Table 5). Figure 3 is an example of a patient showing positional jaw asymmetry after bimaxillary surgery: (a) Maxilla: shift asymmetry (ANS deviation) of −0.96 mm, roll asymmetry of −2.99 degrees, and yaw asymmetry of +1.59 degrees. (b) Mandible: shift asymmetry (mGT deviation) of +2.74 mm, roll asymmetry of +2.53 degrees, and yaw asymmetry of +0.44 degrees. (c) Chin: shift asymmetry (Me deviation) of +2.57 mm, roll asymmetry of +5.41 degrees, and yaw asymmetry of −1.34 degrees. (d) Ramus: shift asymmetry (discrepancy in transverse distances of SPR and SPL from the MSP) of −0.44 mm, roll asymmetry of +2.51 degrees, and yaw asymmetry of −1.21 degrees.

**Table 5:** Variables for evaluation of positional jaw asymmetry.

Variables	Definition
Maxilla	
Shift asymmetry	Transverse distance between ANS and the midsagittal plane (MSP)
Roll asymmetry	Rotation angle of the maxillary central plane (MxCP) from the MSP around y axis, assessed from the frontal view
Yaw asymmetry	Rotation angle of the MxCP from the MSP around z axis, assessed from the top view
Mandible (mandibular body)	
Shift asymmetry	Transverse distance between mGT and the MSP
Roll asymmetry	Rotation angle of the mandibular central plane (MdCP) from the MSP around y axis, assessed from the frontal view
Yaw asymmetry	Rotation angle of the MdCP from the MSP around z axis, assessed from the top view
Chin	
Shift asymmetry	Transverse distance between Me and the MSP
Roll asymmetry	Rotation angle of the chin central plane (ChinCP) from the MSP around y axis, assessed from the frontal view
Yaw asymmetry	Rotation angle of the ChinCP from the MSP around z axis, assessed from the top view
Ramus	
Shift asymmetry	Discrepancy in transverse distances of bilateral sigmoid points from the MSP
Roll asymmetry	Discrepancy in roll angles of bilateral ramal planes (RPs) from the MSP. Roll angle of each RP was the rotation angle from the MSP around y axis, assessed from the frontal view
Yaw asymmetry	Discrepancy in yaw angles of bilateral RPs from the MSP. Yaw angle of each RP was the rotation angle from the MSP around z axis, assessed from the top view

### Reliability

To assess intra-examiner reliability, all CBCT measurements were repeated by the same investigator for 10 randomly chosen patients one month after the initial session. Intra-examiner reliability, analyzed by the intraclass correlation coefficient (ICC), was excellent (mean ICC, 0.962; 95 percent confidence interval, 0.938 to 0.978).

### Statistical analysis

Statistical analyses were performed using the statistical software package SPSS version 19.0 for Windows (SPSS Inc, Chicago, USA). All descriptive statistics are presented as mean ± standard deviation (SD). Paired t test was used to compare the difference in facial asymmetry before and after surgery. To identify the influencing factors for residual asymmetry, stepwise multiple linear regression analysis was used with the postoperative soft tissue and dental asymmetry as the dependent variables and the preoperative soft tissue and dental asymmetry and the postoperative positional asymmetry of the osteotomy segments as the independent variables. To account for multiple comparison, p ≤ 0.01 was considered statistically significant.

## Data Availability

The datasets generated during and/or analysed during the current study are available from the corresponding author on reasonable request.
